# Production and Characterization of Antifungal Compounds Produced by *Lactobacillus plantarum* IMAU10014

**DOI:** 10.1371/journal.pone.0029452

**Published:** 2012-01-19

**Authors:** HaiKuan Wang, YanHua Yan, JiaMing Wang, HePing Zhang, Wei Qi

**Affiliations:** 1 Key Laboratory of Industrial Fermentation Microbiology, Ministry of Education, College of Biotechnology, Tianjin University of Science and Technology, Tianjin, People's Republic of China; 2 Key Laboratory of Dairy Biotechnology and Engineering, Ministry of Education, Inner Mongolia Agricultural University, Huhhot, People's Republic of China; Auburn University, United States of America

## Abstract

*Lactobacillus plantarum* IMAU10014 was isolated from koumiss that produces a broad spectrum of antifungal compounds, all of which were active against plant pathogenic fungi in an agar plate assay. Two major antifungal compounds were extracted from the cell-free supernatant broth of *L. plantarum* IMAU10014. 3-phenyllactic acid and Benzeneacetic acid, 2-propenyl ester were carried out by HPLC, LC-MS, GC-MS, NMR analysis. It is the first report that lactic acid bacteria produce antifungal Benzeneacetic acid, 2-propenyl ester. Of these, the antifungal products also have a broad spectrum of antifungal activity, namely against *Botrytis cinerea*, *Glomerella cingulate*, *Phytophthora drechsleri Tucker*, *Penicillium citrinum*, *Penicillium digitatum* and *Fusarium oxysporum*, which was identified by the overlay and well-diffusion assay. *F. oxysporum*, *P. citrinum* and *P. drechsleri* Tucker were the most sensitive among molds.

## Introduction

Fungal pathogens cause devastating losses of crops and postharvest fruits throughout the world [1]. Many artificial chemical fungicides have been used to prevent and kill fungi in various environments. However, because of their huge populations and high frequency of mutation, a large amount of pathogenic fungi may easily acquire resistance to frequently used fungicides. Several important chemical fungicides such as anilinopyrimidine, benzimidazoles, demethylation inhibitors (DMI), dicarboximide, phenylpyrrole, Qo respiration inhibitors, and strobilurin, have lost their efficacy against pathogenic fungi in the field [2]. To reduce the risk of crop disease and enhance the safety of food and the environment, new, safer fungicides should be discovered and developed [3]. Using microorganisms to prevent fungal pollution has been sparking and gaining interest during the recent years due to consumers' demand for reducing potential negative effects chemical fungicides may have on the environment [4].

It is well known that some lactic acid bacteria produce metabolites that inhibit the growth of fungi and other species of bacteria [3], [5]. Organic acids such as lactic acid and acetic acid produced by lactic acid bacteria are important antimicrobial compounds, and have been reported to possess antifungal activity [6], [7]. Mauch et al. [8] found that the activity of compounds produced by *Lactobacillus brevis* PS1 was higher at low pH values, i.e. pH<5. Heating and/or proteolytic treatment reduced the inhibitory activity of the cell-free supernatant, and indicated that *L. brevis* produces organic acids and proteinaceous compounds which are active against *Fusarium*. Some researches have reported that *Lactobacillus* can produce antifungal substances such as benzoic acid, methylhydantoin, mevalonolactone [4], [9] and short-chain fatty acids [10]. Magnusson and Schnürer [11] discovered that *L. coryniformis* can produce proteinaceous compounds. And Rouse et al. [12] characterized the antifungal peptides produced by lactic acid bacteria. The trial showed that the antifungal culture has the ability to prevent the growth of mold found in apple spoilage. Dal Bello et al. [13] reported the identification and chemical characterization of four antifungal substances produced by *L. plantarum* FST 1.7, including lactic acid, phenyllactic acid and the two cyclic dipeptides cyclo ((L)-Leu-(L)-Pro) and cyclo ((L)-Phe-(L)-Pro). A study described the antifungal culture as having the ability to retard growth of *Fusarium culmorum* and *Fusarium graminearum* found on breads. Another such study reported the production of the antifungal cyclic dipeptides cyclo (L-Phe-L-Pro) and cyclo (L-Phe-traps-4-OH-L-Pro) by lactic acid bacteria which inhibit the growth of food- and feed-borne filamentous fungi and yeasts in a dual-culture agar plate assay [14].

*L. plantarum* IMAU10014 was isolated for its antifungal properties from Xinjiang koumiss in China. Much research has been conducted on the isolation and characterization of *Lactobacillus* strains from koumiss [15]. Characteristics of the active antifungal substances were tested in the previous study, which indicated that the antifungal production was pH-dependent but stable to heat. After the enzymatic treatment of supernatant with trypsin, neutral protease, and proteinase K, a portion of the antifungal activity was lost. *L. plantarum* IMAU10014 can inhibit the growth of *Alternaria solani*, *Phytophthora drechsleri* Tucker, *Fusarium oxysporum* and *Glomerella cingulated* and *Botrytis cinere*. The possible effect of lactic acid was ruled out [16]. However, little work has been done on purifying the compounds for use against plant pathogenic fungi. The focus of this paper was to purify and identify the antifungal compounds produced by *L. plantarum*, as well as to test their activity against several fungal species.

## Materials and Methods

### Microbial strains and culture conditions

The *Lactobacillus plantarum* culture used in this study was incubated by using the method as described by Wang et al. [16]. One loopful of the stock culture of *L. plantarum* IMAU10014 was streaked onto Man Rogosa Sharpe broth (MRS, Fluka) agar plate and incubated at 37°C for 48∼72 h. A single colony representative was selected from the agar plates and examined microscopically, then was preserved in the same medium containing 15% glycerol at −70°C.

*Lactobacillus* seed culture was prepared in a 100 mL flask with 70 mL MRS medium incubated at 37°C for 18 h. A 3% volume of seed culture was used as its inoculant for the flask culture. The flask cultures were carried out in a 250 mL flask with 200 mL MRS medium at 37°C for 30 h.

The strain of *Phytophthora drechsleri* Tucker from the Institute of Plant Protection, Tianjin, PR China was used as the indicator fungus. As a serious pathogen of tomatoes and melons,it appears to be highly sensitive to LAB antifungal compounds. It grew on a potato dextrose agar at 28°C for 5 to 7 days stored at 4°C, and was stored in medium containing 15% glycerol at −70°C and maintained as frozen stocks in a bio-freezer for a considerable amount of time. *Botrytis cinerea*, *Glomerella cingulate*, *Penicillium citrinum*, *Penicillium digitatum* and *Fusarium oxysporum* were used in this study. Molds were grown individually at 28°C for 5 to 7 days and stored at 4°C. Spore inoculum was prepared by growing the molds on PDA slants until the occurrence of sporulation. The spores were then collected by vigorous shaking with sterile water containing 0.1% Tween 80. The spore concentration was determined using a hemocytometer and adjusted to 10^6^ spores per milliliter.

### Determination of antifungal activity

The test substance's inhibition was determined by using a method as described by a Poison Food Technique and the inhibition to the mycelial growth of fungi was assayed [16]. PDA was used as the medium for the test fungus. The media incorporating test compounds at a serial concentration of 1%∼10% (v/v) was inoculated with agar discs of the test fungi (5 mm) at the center. Replicate plates were incubated at 27±2°C for all test fractions. Control plates containing media mixed with sterile water (10%, v/v) were included. After an incubation period of 2 to 6 days, the mycelial growth of fungi (mm) in both treated (T) and control (C) petri dishes was measured diametrically in perpendicular directions until the fungi growth in the control dishes was almost complete. The percentage of growth inhibition (*I*) was calculated using the formula: *I* (%)×*[(C−T)/C]*×100. The corrected inhibition (*IC*) was then calculated as follow: *IC* (%) = *[(C−T)/(C−C_0_)]*×100. C_0_ means the diameter of the test fungi agar discs (5 mm).

### Production of antifungal substance

Fermentations were carried out in a 30 L fermenter containing MRS medium while stirring (50 r/min) at 37°C for 48 h. The production medium was seeded with 5% (v/v) inoculum from an overnight culture of *L. plantarum* IMAU10014. The cell-free supernatant was prepared via centrifugation (8,000 g, 10 min and 4°C), and the supernatants were then filtered through a 0.45-µm-pore-size disposable sterile filter unit to yield the cell-free supernatant [17]. Subsequently, the cell-free supernatant was used to purify the antifungal compounds. Un-inoculated MRS broth was used as a negative control, fractionated, and evaluated in the biotests using the same procedure as the isolated compounds, and all fractions from the purification process were evaluated for antifungal activity after concentration under vacuum and by freeze-drying [14] or rotary evaporator.

### Purification of antifungal compounds

The supernatant was concentrated through freeze-drying. The broth sample weighed 18 g after being freeze-dried and held a metered volume of 100 mL with sterile water. Then 20 mL portions were extracted using petroleum ether, cyclohexane, dichloromethane, ethyl acetate or n-butanol as an extraction solvent (1∶3, v/v); this step was repeated three times. Extracted hydrofacies were concentrated by rotary evaporator under a vacuum at a temperature lower than 40°C, respectively. The partially isolated compounds were found to display antifungal activity and used for further purification.

The active fraction (concentrated hydrofacies phase) was further purified by silica gel column chromatography. A stepwise elution was carried out using a mixture of petroleum ether and anhydrous alcohol (9∶1 and 4∶1, v/v). The isolation of antifungal compounds obtained after fractional collection (two partial) were detected with ultraviolet wavelength of 254 nm on thin layer plates. The antifungal compound obtained after fractional collection merger was then evaporated to dryness. The dried fractions were dissolved in their solvents and assayed for antifungal activity, respectively.

The active part was further purified by preparative RP-HPLC. That was performed on a Agilent 1200 HPLC system (Agilent Technologies, Palo Alto, CA) using a PrepHT C18 column (21.2×150 mm, 5 µm; Agilent 300SB, USA). The elution was monitored using a UV detector at 210 nm. The mobile phases were: (A) acetonitrile; (B) water with 0.1% TFA. The active fraction was eluted with a linear gradient of solvent A increasing from 3% (v/v) to 50% (v/v) in 40 min followed by 10 min at 100% acetonitrile, with a flow rate of 10 ml min^−1^, and the column temperature was 35°C. All peak fractions were collected. Before determining whether or not antifungal activity was present, the acetonitrile was evaporated by incubation at 40°C for 24 h and the fractions were freeze-dried [17]. The partially purified compounds were used for further structural analysis. Reference samples were prepared with commercial 3-phenyllactic acid.

### Identification of the antifungal compounds

Active fraction I was analyzed by liquid chromatography–mass spectrometry (LC-MS) which was performed on an UPLC system and an ESQUIRE-LC quadrupole ion trap mass spectrometer (Bruker Daltonic, CA, USA) with electrospray ionization. For chromatographic separation, a BEH C18 column (50×2.1 mm, 1.7 µm; Thermo Fisher Scientific) was used at 25°C. The mobile phases were: (A) acetonitrile with 0.1% formic acid; and (B) water with 0.1% formic acid. The active fraction I was eluted with a linear gradient of solvent A increasing from 20% to 80% at a flow rate of 0.3 ml min^−1^ for 12 min. The HPLC output was split to a reduced flow rate of 0.2 ml min^−1^ before entering the electrospray ionization source. The mass scan range was m/z 50–1800. The active fraction II was analyzed on **a** GC-MS instrument. An aliquot (<1 mg) of dry sample was dissolved in 1 ml of dichloromethane. The GC injector was kept at 80°C, and the GC-MS interface was kept at 280°C. Helium was used as the carrier gas with a flow of 1 ml min^−1^.

The structure of the antifungal compounds was determined using nuclear magnetic resonance (NMR). NMR spectra were recorded on a spectrometer equipped with a 2.5 mm microprobe using dimethylsulphoxide-d6 as a solvent. All spectra were recorded at 30°C. Chemical shifts are reported relative to the solvent peaks (dimethylsulfoxide-d6 ^1^H δ2.50 and ^13^C δ40.02).

### Determination of the inhibition spectrum of activity compounds

The inhibition spectrum of test compounds was determined by using the overlay and well-diffusion assay with a few modifications and the inhibition to the spore growth of pathogenic fungi was assayed, as well. Ten milliliters of 2% agar was poured into a sterile petri dish and allowed to harden. The plates were overlaid with potato dextrose soft agar containing 10^6^ spores per milliliter. Wells (7 mm diameter) were punched in the plates using a sterile stainless steel borer. The wells, have been bored into the test dishes, were filled with 180 uL of the sample and allowed to diffuse in to the agar during a 5 h pre-incubation period at room temperature followed by incubation at 27±2°C for 48 h. Then the inhibition zone around the sample was measured. The indicator fungi were *B. cinerea*, *A. solani*, *P. drechsleri Tucker*, *P. citrinum*, *P. digitatum* and *G. cingulata*. The inhibition was graded according to the size of inhibited growth area. The following size was used: −, no visible inhibition; +, 0.1∼7 mm zone of inhibition; ++, 7.1∼14 mm zone of inhibition; +++, >14 mm zone of inhibition.

## Results and Discussion

### Activity against *Phytophthora drechsleri* Tucker of *Lactobacillus plantarum* IMAU10014

**Figure 1:**
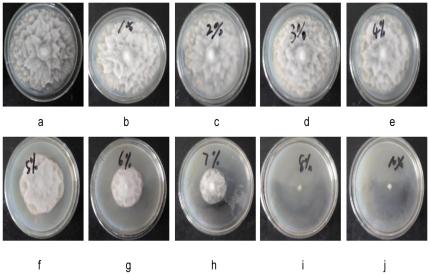
The antifungal activity of *L. plantarum* IMAU10014 cell-free supernatant against *P. drechsleri* Tucker. The Potato dextrose agar plates incorporating the supernatant of *L. plantarum* IMAU10014 at a concentration of 1%∼10% (b: 1%; c: 2%; d: 3%; e: 4%; f: 5%; g: 6%; h: 7%; i: 8%; j: 10% (v/v)) was inoculated with agar discs of the test fungi (5 mm) at the center. a: (control plate) containing media mixed with sterile water (10%, v/v) was inoculated with agar discs of the test fungi (5 mm) at the center.

Among the fungal cultures tested, minimum inhibitory concentration value against fungal cultures was 3% (v/v). The media incorporating cell-free culture at a concentration of 8%∼10% (v/v), 100% inhibition was shown while using *P. drechsleri* Tucker as indicator strain in Figure 1. The result revealed that *L. plantarum* IMAU10014 had marked inhibitory capacity against *P. drechsleri* Tucker.

Sjogren et al. [10] reported that several of the strains showed strong inhibitory activity against the moulds *A.fumigatus*, *Aspergillus nidulans*, *Penicillium commune* and *Fusarium sporotrichioides*, and also against the *yeast Rhodotorula mucilaginosa*. Prema et al. [4] showed that *L. plantarum* strain displays an inhibitory effect against a broad range of filamentous fungi. Most of these fungal species are spoilage organisms found in baked-goods.

LAB with antifungal activity holds the potential to be used as a natural fungicide, as it prevents the growth of pathogen in certain plant species. But there are few reports on the activity against plant pathogenic fungi and the antifungal principles characteristics of *L. plantarum* that we know of thus far [16]. *Phytophthora* are pathogens that infect a wide range of plant species. For dicot hosts such as the tomato, potato and soybean, *Phytophthora* could even be considered as the most important of pathogens [18]. In any case, this is a really exciting result for fungicides in the market, because it is environmentally safe and displays high thermal stability.

### Purification of the antifungal compounds

The organic and aqueous phase were obtained through Liquid-liquid extraction. The results showed that the antifungal material exists largely in the aqueous phase, which was extracted by petroleum ether and cyclohexane. N-butanol itself can inhibit hypha growth of *P. derchsleri* Tucker and ethyl acetate extract showed strong activity, as well. The percentage of growth inhibition (*I*) was 63.7% (Table 1).

**Table 1:** Mean % inhibition (±SD) by extraction phase and raffinate phase against *P. derchsleri* Tucker.

	extraction phase (I%)	raffinate phase (I%)
petroleum ether	1.2±0.9	97.3±1.7
cyclohexane	1.6±1.4	98.4±2.2
dichloromethane	33.5±2.2	58.4±2.9
ethyl acetate	63.7±1.9	12.2±4.3
n-butanol	99.6±0.6	100.0±0.0

**Figure 2:**
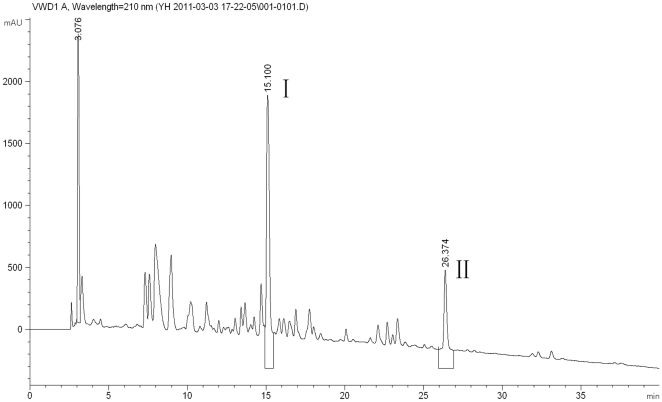
HPLC profile of antifungal compounds produced by *L. plantarum* IMAU10014.

The antifungal substance of ethyl acetate extracts was loaded into a silica gel column. The isolation of antifungal compounds obtained after fractional collection (two partial) were detected with an ultraviolet wavelength of 254 nm on thin layer plates. The A2 partial showed strong antifungal activity, however, the A1 partial had no effect (Table 2). The active partial was further purified by preparative reverse phase HPLC with a C18 column based on hydrophobicity. Two active fractions were obtained, at last. Two of the antifungal compounds eluted at retention times of 15.10 and 26.37 min were isolated and designated as I and II, respectively (Figure 2). Data from the experiments elucidating the chemical structures of compound I and II is summarized below.

**Table 2:** Mean % inhibition (±SD) by sample A1 and A2 against *P. derchsleri* Tucker.

Sample	I%
Control	0±0.8
A1	0±2.1
A2	86.9±1.7

Spiking experiments with commercial, pure substances followed by an analytical HPLC analyses led to the identification of 3-phenyllactic acid (relative retention time of 15.08 to 15.13) (Table 3).

**Table 3:** HPLC analyses of 3-phenyllactic acid isolated from cell-free supernatants of *L. plantarum* IMAU10014.

Relative retention time/min			
Analyte	Reference	Spiked cell-free supernatant	Cell-free supernatant
3-Phenyllactic acid	15.13	15.08	15.10

### Analysis of active compounds

**Figure 3:**
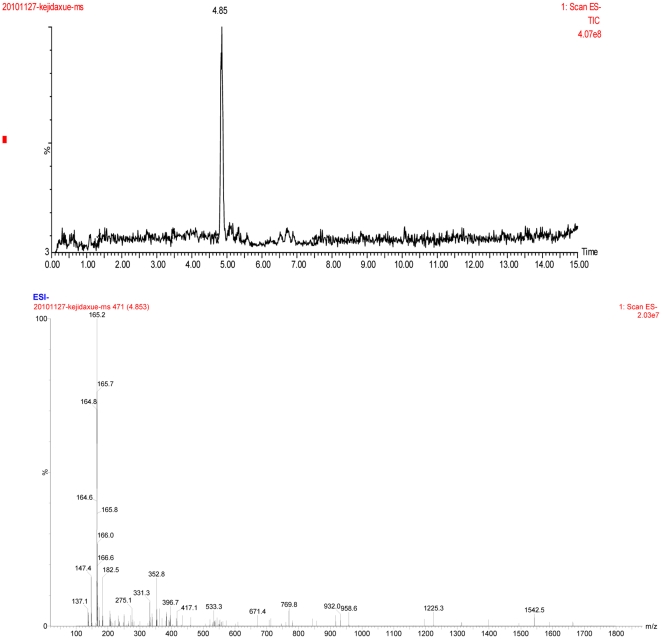
LC-MS profile of antifungal compound I produced by *L. plantarum* IMAU10014.

**Figure 4:**
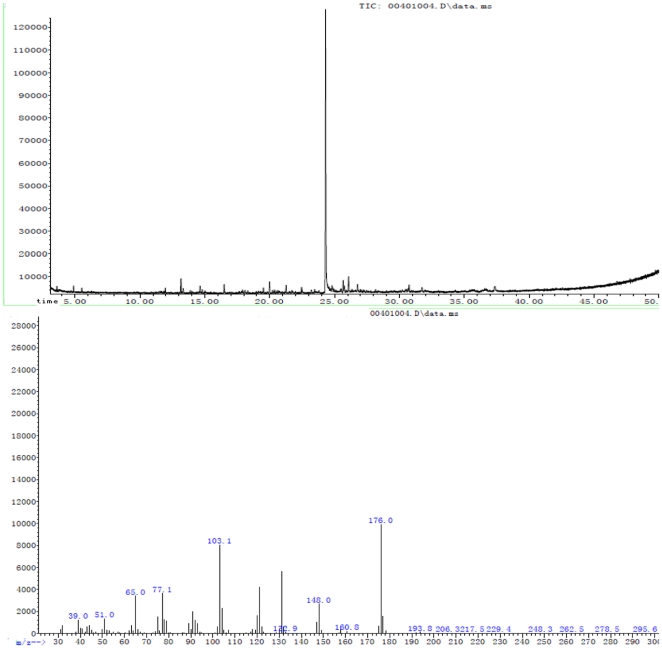
GC-MS profile of antifungal compound II produced by *L. plantarum* IMAU10014.

From a total of 5 L of culture, approximately 17 mg of I and 12 mg of II were obtained. These two active fractions were then subjected to ESI–MS analysis. Basing on the [M+H]^+^ ions, the molecular masses of I and II were determined to be 165 and 176, respectively (Figure 3 and Figure 4). For compound I: HPLC retention time tR (min): 15.10 (standard 15.08); ESI-MS (m/z): 165 (M+H)^+^; ^1^H NMR (400 MHz, DMSO-d6); δ7.31 (H-5 and H-9), 7.25 (H-6 to H-8), 4.16 (H-2), 2.95 (H-3a), 2.82(H-3b); ^13^C NMR (400 MHz, DMSO-d6); δ175.59 (C-1), 71.52 (C-2), 40.47 (C-3), 138.60 (C-4), 128.43 (C-5 and C-9), 129.85 (C-6 and C-8), 126.57 (C-7); The ligands formed for the produced compound were similar to the ligands of the commercial 3-phenyllactic acid. For compound II, solid; ^13^C NMR (400 MHz, DMSO-d6); δ171.34 (C-1), 46.12 (C-2), 134.81 (C-3), 129.83 (C-4 and C-8), 129.22 (C-5 and C-7), 127.65 (C-6), 67.53 (C-1′), 133.62 (C-2′), 116.35 (C-3′); ESI-MS (m/z): 176 (M+H)^+^; HPLC retention time tR (min): 26.37. Compound II was identified as Benzeneacetic acid, 2-propenyl ester by comparing data from NMR, ESI-MS, and HPLC.

The spectrum of antifungal activity using the two commercial compounds displayed better antifungal activity against six fungal strains (Table 4). For 3-phenyllactic acid, *F. oxysporum* and *P. drechsleri* Tucker were the most sensitive among the molds, followed by *B. cinerea*, *G. cingulate*, *P. digitatum*, which were more sensitive compared to *P. citrinum*, which exhibited less sensitivity. For Benzeneacetic acid, 2-propenyl ester, *P. citrinum* and *P. drechsleri* Tucker were the most sensitive among the molds, followed by *F. oxysporum*, *B. cinerea*, *G. cingulate*, *P. digitatum*, which were more sensitive.

**Table 4:** Antifungal activity spectrum of antifungal compounds against the spore growth of selected fungal cultures.

Pathogens	3-phenyllactic acid	Benzeneacetic acid, 2-propenyl ester
*P. drechsleri* Tucker	+++	+++
*G. cingulate*	++	++
*B. cinerea*	++	++
*P. citrinum*	+	+++
*P. digitatum*	++	++
*F. oxysporum*	+++	++

− no visible inhibition.

+ 0.1∼7 mm zone of inhibition.

++ 7.1∼14 mm zone of inhibition.

+++ >14 mm zone of inhibition.

A series of studies on LAB indicated that some of the antifungal activity was caused by organic acids, proteinaceous compounds or other end products [19], [20]. Strom et al. [14] reported that cyclic dipeptides and 3-phenyllactic acid from *Lactobacillus plantarum* strain (MiLAB 393) were purified on a C18 SPE column and a HPLC C18 column, and then were verified by nuclear magnetic resonance spectroscopy, mass spectrometry, and gas chromatography. Niku-Paavola et al. [9] identified benzoic acid, 5-methyl-2,4-imidazolidinedione, tetrahydro-4-hydroxy-4-methyl-2H-pyran-2-one and 3-(2-methylpropyl)-2,5-piperazinedione from *L. plantarum* VTTE-78076 against gram-negative test organism, *Pantoea agglomerans* VTTE-90396 and *F. avenaceum* VTTD-80147. Prema et al. [4] and Lavermicocca et al. [21], antifungal compound produced by *L. plantarum* strain displayed growth inhibition against common fungal strains such as *A. niger*, *A. fumigatus*, *A. terreus*, *A. flavus*, *A. nidulans*, *P. roquefoti*, *P. corylophilum*, *P. expansum*, and *P. camembertii*.

Some antifungal compounds have been successfully purified. however, most of these compounds remain to be identified due to the lack of suitable assay procedures to isolate small amounts of active metabolites. Therefore, further studies should be devoted to the development of efficient methods for detecting antifungal compounds in complex biological systems and controlling optimal conditions responsible for better antifungal metabolite production in vitro [22]. The presence of these antifungal compounds and other unidentified substances should be evaluated in greenhouse tests to further elucidate the protective effect on crops.

### Conclusion

In the agar test system used for determination of antifungal activity in this study, *Lactobacillus plantarum* IMAU10014 showed strong activity against *Phytophthora. drechsleri* Tucker. Two of the fungal inhibitory substances were purified from the *L. plantarum* IMAU10014. The production of the antifungal Benzeneacetic acid, 2-propenyl ester by lactic acid bacteria is reported here for the first time. Furthermore, the antifungal mechanism of *L. plantarum* IMAU10014 and putative synergistic effects in vitro are proposed as follows.
